# Appendiceal band syndrome causing acute small bowel obstruction with concomitant appendicitis: a case report

**DOI:** 10.1093/jscr/rjag452

**Published:** 2026-06-10

**Authors:** Abdisalam Ismail Hassan, Hodan Abdi Hassan, Abdinasir Mohamed Elmi, Abdirahman Ahmed Omar, Ahmed Muhammad Bashir, Nuradin Mohamed Nur

**Affiliations:** Faculty of Medicine and Health Sciences, Jamhuriya University of Science and Technology, Mogadishu, Somalia; Obstetrics and Gynecology Department, Mogadishu Somali–Türkiye Recep Tayyip Erdoğan Training and Research Hospital, Mogadishu, Somalia; Radiology Department, Mogadishu Somali–Türkiye Recep Tayyip Erdoğan Training and Research Hospital, Mogadishu, Somalia; General Surgery Department, Mogadishu Somali–Türkiye Recep Tayyip Erdoğan Training and Research Hospital, Mogadishu, Somalia; Internal Medicine Department, Somali Sudanese Specialized Hospital, Mogadishu, Somalia; General Surgery Department, Mogadishu Somali–Türkiye Recep Tayyip Erdoğan Training and Research Hospital, Mogadishu, Somalia

**Keywords:** appendiceal band syndrome, appendicular knot, small bowel obstruction, acute appendicitis, case report

## Abstract

Appendiceal band syndrome is a rare complication of acute appendicitis in which the appendix encircles a segment of small bowel, causing mechanical obstruction. Due to its rarity and nonspecific presentation, diagnosis is often made intraoperatively, and delayed recognition may lead to bowel strangulation. A 23-year-old male presented with acute abdominal pain, vomiting, distension, and obstipation, with no prior surgical history. Computed tomography showed dilated small bowel loops with a distal ileal transition point, an inflamed appendix, and mesenteric twisting. Emergency laparotomy confirmed small bowel obstruction due to an appendiceal band with acute appendicitis. Adhesiolysis and appendectomy were performed, leading to complete resolution. The postoperative course was uneventful. Appendiceal band syndrome should be considered in young patients with obstruction and no prior surgery. Early surgical intervention is essential for favorable outcomes.

## Introduction

Appendiceal band syndrome, also known as appendicular knot syndrome, appendicular tie syndrome, appendico-ileal knot, or appendiceal tourniquet, is an extremely rare surgical emergency [1, 2]. It occurs when the appendix forms a loop through which a segment of bowel becomes entrapped [1]. An inflamed and elongated appendix may adhere to adjacent structures such as the cecum, small intestine, or mesentery, creating a constricting ring that leads to mechanical small bowel obstruction and, in severe cases, bowel strangulation and ischemia [3]. Only a limited number of cases have been reported, mostly as isolated case reports.

Intestinal obstruction is a common and potentially life-threatening emergency, accounting for 2%–8% of acute abdomen presentations, with the small intestine involved in nearly 80% of cases [1, 3]. Common causes include adhesions, hernias, and neoplasms [1, 3]. Appendiceal band syndrome is pathophysiologically similar to, but clinically distinct from, postoperative adhesions involving the appendiceal stump [2].

Preoperative diagnosis is challenging due to its rarity and nonspecific presentation, and most cases are diagnosed intraoperatively. Delayed recognition may result in complications such as strangulation, ischemia, and perforation [2]. We report a rare case of appendiceal band syndrome causing acute small bowel obstruction with concomitant appendicitis in a young adult with no prior abdominal surgery, emphasizing the importance of early recognition and timely surgical intervention.

## Case presentation

A 23-year-old male presented with acute severe abdominal pain, recurrent vomiting, progressive abdominal distension, and obstipation. The pain was diffuse, colicky, and progressively worsening, with inability to tolerate oral intake. Vomiting was non-bilious and non-bloody.

There was no history of fever, hematemesis, melena, jaundice, urinary symptoms, trauma, or similar prior episodes. He had no history of abdominal surgery or chronic illness and was not on regular medication. Family and social history were unremarkable.

On examination, the patient appeared distressed but was alert. Vital signs showed mild tachycardia, with other parameters normal. The abdomen was distended with diffuse tenderness, more pronounced in the lower abdomen, and associated with guarding but no rebound tenderness. No masses or hernias were detected. Bowel sounds were initially hyperactive and later reduced. Rectal examination revealed an empty rectum. Other systemic examinations were normal.

**Figure 1:**
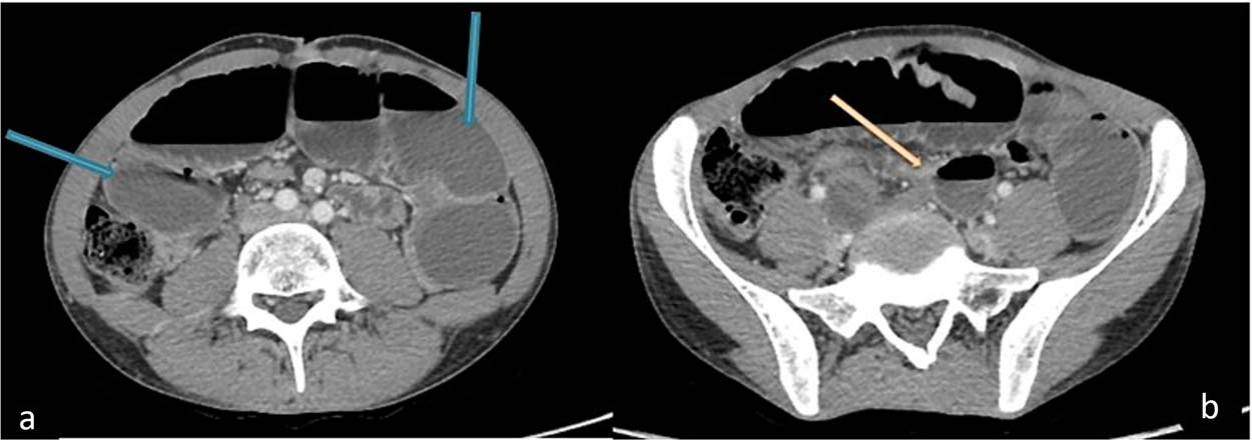
(a and b) Dilated proximal small bowel loops are observed (Fig. 1a, blue arrows), with a distinct transition point at the distal ileum where the bowel caliber abruptly changes to collapsed distal loops (Fig. 1b, orange arrow).

**Figure 2:**
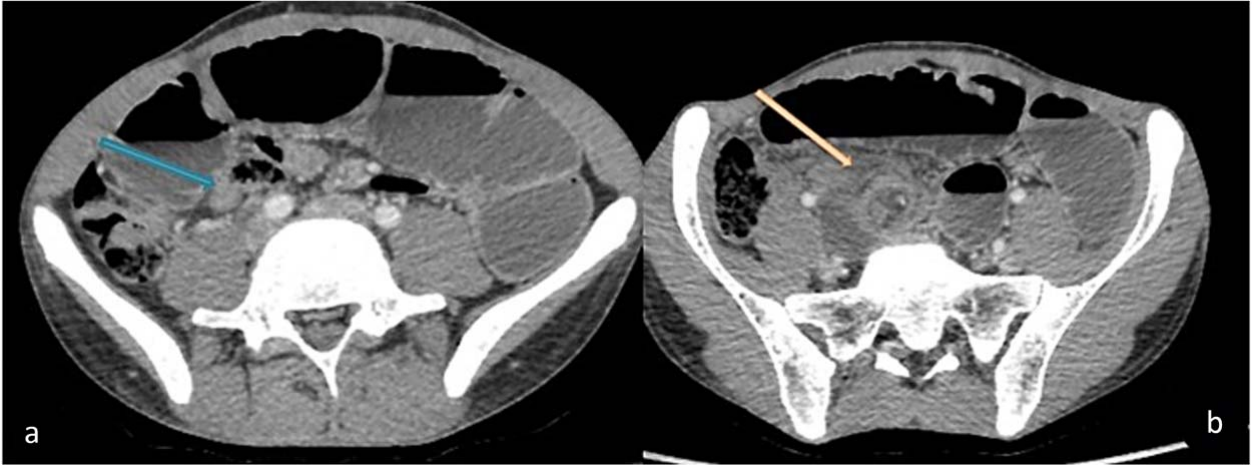
(a and b) A dilated appendix with surrounding inflammation is demonstrated (Fig. 2a, blue arrow), with associated twisting of the mesenteric vessels in the right lower quadrant (Fig. 2b, orange arrow).

Plain abdominal radiography showed dilated small bowel loops with air–fluid levels. Contrast-enhanced computed tomography (CT) demonstrated dilated proximal small bowel loops (Fig. 1) with a transition point at the distal ileum (Fig. 1b), confirming obstruction. It also revealed a dilated inflamed appendix (Fig. 2) and twisting of mesenteric vessels in the right lower quadrant (Fig. 2b), suggesting concurrent appendicitis.

**Figure 3:**
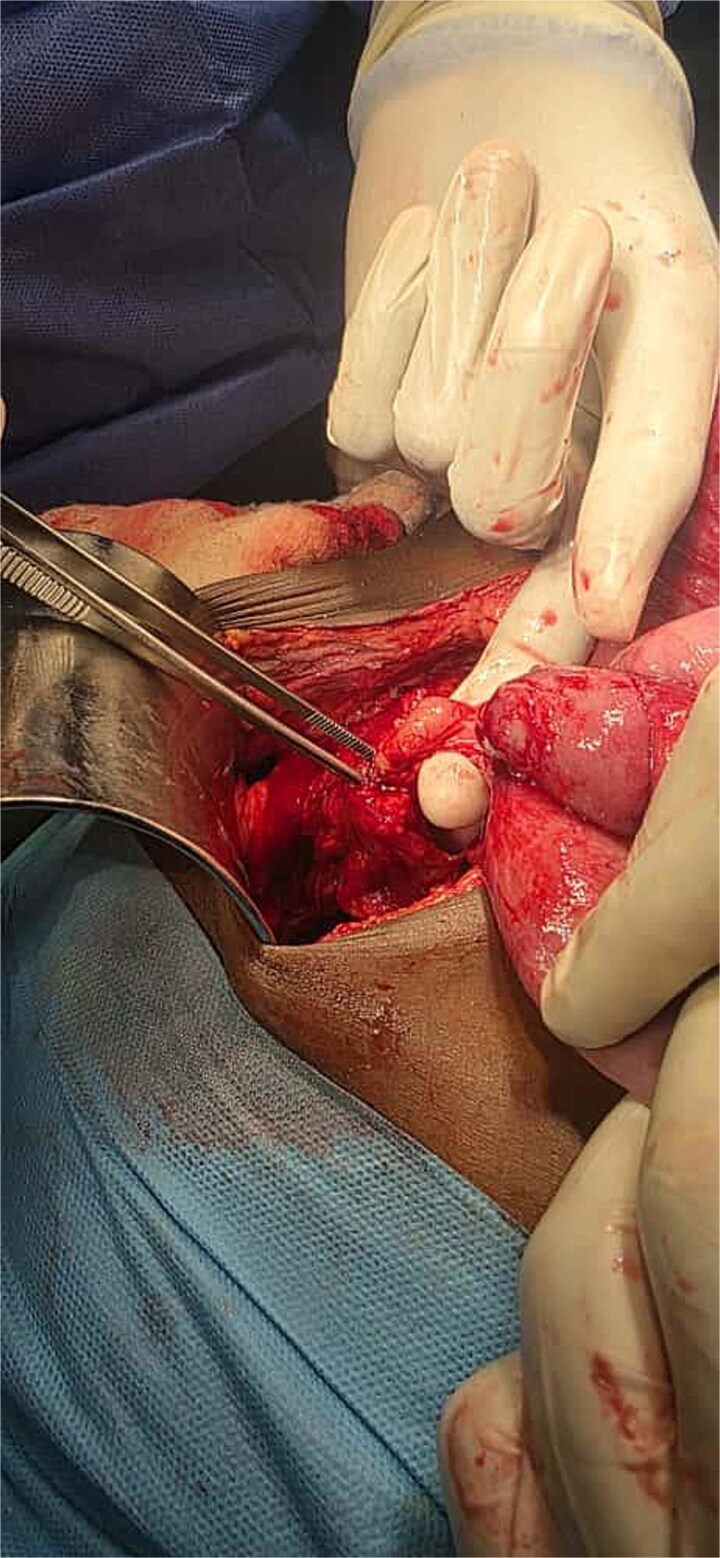
Intraoperative view showing an appendiceal band encircling a loop of small bowel, causing mechanical intestinal obstruction.

**Figure 4:**
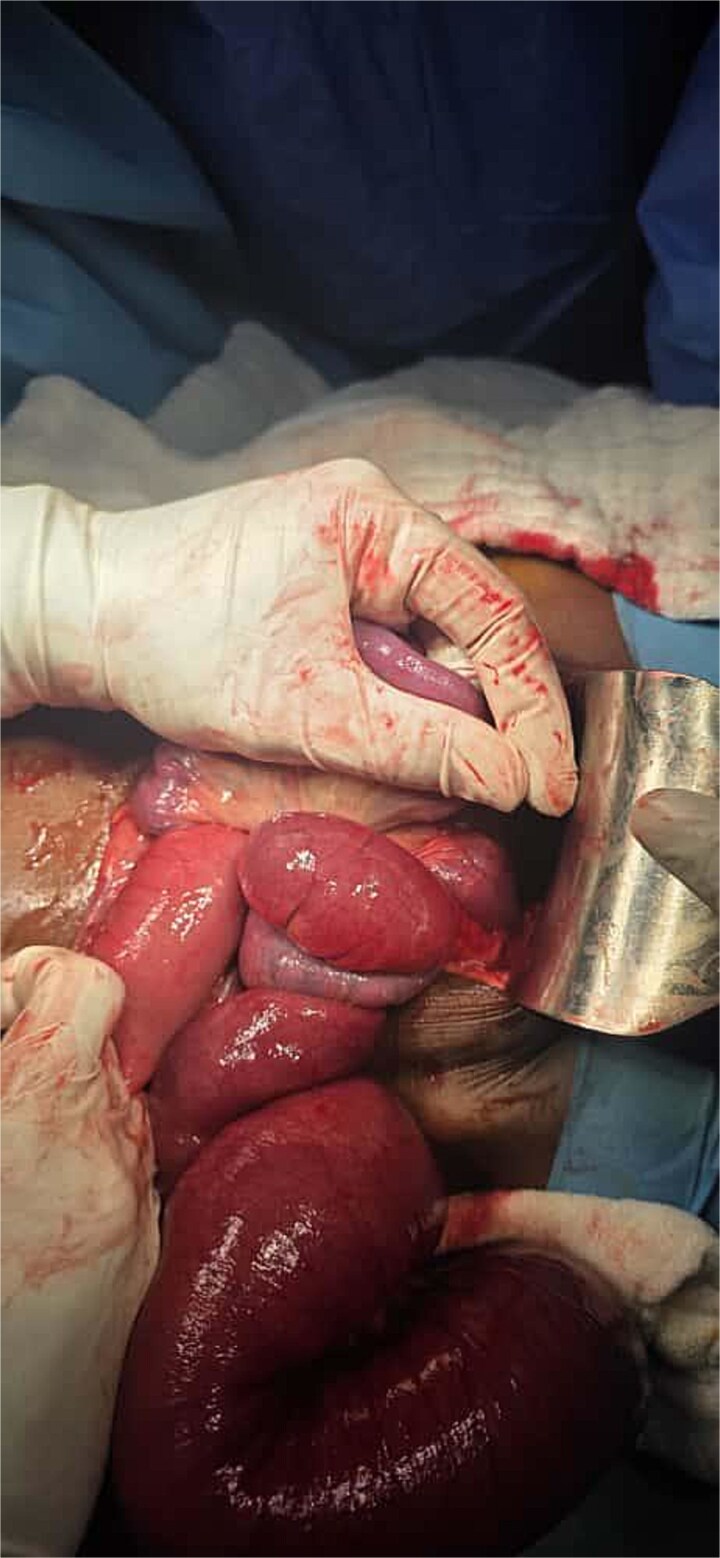
Intraoperative view showing proximal small bowel dilatation with distal bowel collapse, consistent with mechanical small bowel obstruction.

A diagnosis of small bowel obstruction associated with acute appendicitis was made, and emergency laparotomy was performed. Intraoperatively, distal small bowel obstruction due to adhesions with twisted loops was identified. Adhesiolysis relieved the obstruction, and appendectomy was performed (Figs 3 and 4). Hemostasis was achieved with minimal blood loss, and drains were placed.

The postoperative course was uneventful. The patient resumed oral intake on Day 2 and was discharged on Day 3. Follow-up at 1 week, 1 month, and 6 months showed complete recovery without recurrence.

## Discussion

Appendiceal band syndrome (ABS), also known as appendicular knot or appendiceal tourniquet, is a rare but potentially life-threatening cause of mechanical small bowel obstruction. It occurs when an inflamed or elongated appendix adheres to adjacent structures such as the ileum, mesentery, or cecum, forming a loop that entraps bowel [3, 4]. This can result in closed-loop obstruction with rapid progression to strangulation, ischemia, and perforation if untreated [5, 6].

First described by Hotchkiss in 1901 [7], ABS remains rare and is mainly reported as isolated case reports or small series [3, 7]. It typically occurs in patients without prior abdominal surgery, distinguishing it from postoperative adhesions [5, 8]. This was evident in our patient with a “virgin abdomen,” narrowing the differential diagnosis.

Clinically, ABS presents with nonspecific features of small bowel obstruction—abdominal pain, vomiting, distension, and obstipation—which may obscure underlying appendiceal pathology [8, 9]. In such cases, especially in young patients without surgical history, rare causes such as congenital bands, internal hernias, volvulus, and inflammatory conditions like ABS should be considered [10].

Contrast-enhanced CT is the imaging modality of choice and provides important diagnostic clues, including dilated bowel loops, a transition point, mesenteric twisting, and appendiceal inflammation [3, 4]. Although definitive preoperative diagnosis is uncommon, these findings should raise suspicion of a complex obstructive process [11, 12]. As in most cases, diagnosis in our patient was confirmed intraoperatively.

Surgical intervention is the definitive treatment and should not be delayed. Management includes release of the appendiceal band, adhesiolysis, and appendectomy [5, 6]. Early intervention prevents complications such as ischemia and gangrene that may require bowel resection [13, 14]. In our case, timely surgery resulted in complete recovery without complications.

From a pathophysiological perspective, ABS resembles other intestinal knotting syndromes, such as ileo-ileal and ileo-sigmoid knotting, where closed-loop obstruction compromises blood flow and accelerates necrosis if untreated [10]. Failure to recognize ABS may lead to delayed diagnosis and increased morbidity. The favorable outcome in our patient highlights the importance of early surgical management.

## Conclusion

ABS is a rare but serious cause of mechanical small bowel obstruction, particularly in patients without prior abdominal surgery. Its presentation often mimics more common causes, making diagnosis challenging. Contrast-enhanced CT can provide useful clues, but definitive diagnosis is usually intraoperative. Early surgical exploration with release of the appendiceal band and appendectomy is essential to prevent complications such as ischemia and perforation. Increased awareness among clinicians is crucial for timely diagnosis and improved outcomes.

## Data Availability

All relevant data supporting the findings of this case report are included within the article. Additional details are available from the corresponding author on reasonable request.
